# A Systematic Review of Biopsychosocial Training Programs for the Self-Management of Emotional Stress: Potential Applications for the Military

**DOI:** 10.1155/2013/747694

**Published:** 2013-09-23

**Authors:** Cindy Crawford, Dawn B. Wallerstedt, Raheleh Khorsan, Shawn S. Clausen, Wayne B. Jonas, Joan A. G. Walter

**Affiliations:** ^1^Samueli Institute, 1737 King Street, Suite 600, Alexandria, VA 22314, USA; ^2^Samueli Institute, 2101 East Coast Highway, Suite 300, Corona Del Mar, CA 92625, USA; ^3^Walter Reed National Military Medical Center, 8901 Wisconsin Avenue, Building 8, Room 5106, Bethesda, MD 20889, USA

## Abstract

Combat-exposed troops and their family members are at risk for stress reactions and related disorders. Multimodal biopsychosocial training programs incorporating complementary and alternative self-management techniques have the potential to reduce stress-related symptoms and dysfunction. Such training can preempt or attenuate the posttraumatic stress response and may be effectively incorporated into the training cycle for deploying and redeploying troops and their families. A large systematic review was conducted to survey the literature on multimodal training programs for the self-management of emotional stress. This report is an overview of the randomized controlled trials (RCTs) identified in this systematic review. Select programs such as mindfulness-Based Stress Reduction, Cognitive Behavioral Stress Management, Autogenic Training, Relaxation Response Training, and other meditation and mind-body skills practices are highlighted, and the feasibility of their implementation within military settings is addressed.

## 1. Introduction

Combat-exposed troops and their family members are at risk for stress reactions and related disorders [1]. Strategies to enhance psychological resilience among service members are needed. Providing this training prior to deployment might preempt or attenuate the posttraumatic stress response, depression, anxiety, and other consequences of overwhelming stress.

Complementary and alternative medicine (CAM) and integrative medicine (IM) approaches to self-management of emotional stress are increasingly utilized within comprehensive care models [2]. Surveys have affirmed the widespread use of integrative modalities in military populations and settings, including Department of Defense (DoD) beneficiaries [3], active duty military [4], and patients using Veterans Health Administration (VHA) hospitals [5–7].

Multimodal treatment programs, as compared to single modality treatments, have emerged as an important option in the management of stress disorders [8, 9]. Compared to treatment with a single modality, multimodal programs have the potential to simultaneously address a range of stress reactions, both physical and mental, as well as the dynamic nature of the disease process over time. Applied at the population level, the increased variety of modalities potentially has a greater chance of providing viable alternatives for a given individual.

The military is already a culture in which self-care is recognized as a vital tool in warfare: adequate nutrition, hydration, and sleep are part of a warrior's battle kit. Therefore, self-management skills that are delivered as multimodal programs involving CAM/IM may be an ideal option for the military community to help build resilience, reset the autonomic nervous system, and ease emotional stress. Consequently, in tandem with the mainstreaming of many CAM practices in the civilian sector, military personnel may seek CAM therapies to improve their health and well-being, especially those CAM therapies that are self-manageable and drugless.

A number of model biopsychosocial training programs exist in the civilian sector [10–13]. Several have adapted their programs to the military [10, 12–14], and a smaller number have provided training to military personnel [14]. However, to date, there has been no comprehensive systematic review of multimodal biopsychosocial programs for the self-management of stress. The authors posit that these interventions could be markedly effective for individuals in high-stress environments such as the military.

### 1.1. Purpose of the Review

A comprehensive systematic review was conducted to (1) survey multimodal biopsychosocial training programs with at least one CAM/IM component for the self-management of emotional stress across all populations; (2) assess the quantity and quality of the research and programs; and (3) characterize the results by type of program and strength of evidence on stress-related outcome measures. Due to the massive amount of the literature included, the full report of this review will be detailed in a much larger report, not suitable for detailing in one paper. The purpose of this current report is to (1) focus on those studies that were scored as high quality and demonstrated statistically significant results between groups (i.e., intervention group versus control or comparison group) on outcomes of emotional stress for controlled trial study designs; (2) describe the multimodal programs available and their characteristics; (3) describe the results of outcomes related to emotional stress; and (4) discuss what the authors believe to be the resource requirements needed to incorporate these programs into a military setting for service members and their families. The authors have chosen to report this subset of data since studies assessed as high quality according to internal validity criteria are the least likely to have introduced bias, are more likely replicable, and can be trusted to show a valid effect for the intervention and population being studied [15, 16]. If these studies are generalizable to other populations, then it is reasonable to anticipate that an effective program, if implemented in a military environment, could show equivalent benefits for this highly stressed population.

## 2. Methods

### 2.1. Concepts and Definitions

#### 2.1.1. Biopsychosocial Model

The biopsychosocial model (abbreviated “BPS”) is a term introduced in 1977 by the American psychiatrist George Engel which describes a health care perspective that acknowledges that biological, psychological (which entails thoughts, emotions, and behaviors), and social factors all play a significant role in human functioning in the context of wellness and illness. It is a term that is often used to describe the concept of the “mind-body connection” [8].

#### 2.1.2. Complementary and Alternative Medicine (CAM) Modality

CAM is defined at the National Center for Complementary and Alternative Medicine as: any of a number of “diverse medical and health care systems, practices, and products that are not generally considered part of conventional medicine” (http://nccam.nih.gov/). For the purposes of this review, the authors included only CAM modalities that also met our criteria for the biopsychosocial model and self-management technique (http://www.ncbi.nlm.nih.gov/mesh/?term=complementary+therapies) and used definitions of CAM techniques of breathing, relaxation, yoga, imagery, hypnosis, and meditation as described by the National Center for Health Statistics on the NCCAM website: http://nccam.nih.gov/health/providers/camterms.htm.

#### 2.1.3. Self-Management Technique

Self-management techniques are techniques in which skills are used independently by an individual without ongoing reliance on a trainer or therapist. The authors excluded interventions where patients solely learn and integrate therapies by themselves (such as through a book or online material) or ones that are trainer or therapist dependent (i.e., psychotherapy that requires a therapist to lead the sessions).

#### 2.1.4. Multimodality Interventions

These interventions are defined as those ones that have two or more interventions (at least one of which is CAM modality) that require an initial training period with a therapist or trainer in which skills are learned, all of which can be transferred into self-management techniques. The authors only included programs that have multimodal interventions. An example is a program that includes relaxation, exercise, and behavioral techniques to reduce stress. The thought here was that multimodal programs would allow for more of a biopsychosocial approach to treating the whole person for the complexities of emotional stress.

#### 2.1.5. Types of Program

Types of programs that the authors considered were those that include training in at least one self-management multimodal training with the intention to reduce psychological or emotional stress. The program did not necessarily have to be an existing named program per se but had to include interventions that could be developed into a program (e.g., a potential program). The authors included educational training programs as long as they met these criteria.

### 2.2. Search Strategy

The following electronic databases were searched from database inception through February 2009 across keywords identified: PUBMED, EmBase, BIOSIS, CINAHL, the entire Cochrane library as well as the database of abstracts of reviews of effectiveness (DARE), PILOTS, PsycInfo, AMED, ERIC, and DoD Biomedical Research. Gray literature was also searched for unpublished trials via the Register of the Controlled Trials databases (http://www.controlled-trials.com/ and http://www.clinicaltrials.gov/), NLM catalog, and NCCAM Grantee Publications Database, communicating with identified experts in the field of CAM/IM for additional reports of studies not included through traditional searching and pearling references of included articles.

### 2.3. Study Selection

Studies were included if they involved (1) research on a program or potential program; (2) a multimodal intervention incorporating at least one CAM modality (as defined by NCCAM and detailed by the National Library of Medicine (NLM) http://nccam.nih.gov/health/providers/camterms.htm; http://www.ncbi.nlm.nih.gov/mesh/?term=complementary+therapies); (3) skills that were learned that could be used as self-management techniques, after a training period; (4) at least one outcome measure of psychological or emotional stress; (5) human subjects from any clinical or nonclinical population; and (6) were presented in the English language. To encompass the construct of “psychological or emotional stress,” studies were included that used outcome measures containing one of the following keywords: *stress*, *anxiety*, *post-traumatic stress disorder*, *coping*, *resilience*, *hardiness*, *burnout*, *distress*, or *relaxation* at the screening phase. Since all healthy and clinical populations experience stress (although to varying degrees), the authors elected not to exclude any population based on predefined criteria about their conditions or diagnoses. This was consistent with the authors' intent to generalize about the value of these programs impacting the symptoms of stress. Thus, all populations, both healthy and clinical (e.g., those with specific conditions or diagnoses), were included as long as the report included a description of emotional stress as defined above. Types of the literature excluded from this systematic review, were thought pieces, descriptive reviews or published expert opinions. The authors excluded the following interventions: ones in which individuals learned and integrated therapies by themselves; those that involved website training, books, or leaflets as the sole source of the training; pharmacological agents or placebos; and pet therapy. Cognitive behavioral therapy (CBT) was not defined as a CAM practice by itself but was included when it incorporated another CAM technique, such as deep breathing or relaxation exercises that were the predominant feature of the program. All programs had to involve at least one CAM modality as described above; no additional medical or psychosocial procedures were included unless the program integrated those procedures with the CAM modality for the purpose of stress management. See Box 1 for the search terms used.

Five investigators (Cindy Crawford, Sasha Knowlton, Raheleh Khorsan, Dawn Wallerstedt, and Shawn Clausen) individually and independently screened all titles and abstracts in duplicate for relevance based on the inclusion criteria mentioned above. Weekly team meetings were held between all five screeners to resolve any and all disagreements.

#### 2.3.1. Quality Assessment and Data Extraction

The methodological quality of included RCT studies was assessed independently by four reviewers in duplicate using a modified version of the Scottish Intercollegiate Guidelines Network (SIGN 50) checklist, a validated and reliable assessment approach widely used in the literature [17]. Three of the 10 SIGN criteria for assessment of quality were omitted as they did not apply to our research question (see Table 1). High quality was defined as a SIGN 50 score equal to + (only 1-2 criteria scored as poorly addressed) or ++ (0 criteria scored as poorly addressed); that is, some or all of the internal validity criteria have been fulfilled. Where they have not been fulfilled, the conclusions of the study were thought unlikely to very unlikely to alter results [17]. All reviewers were fully trained in the methodology employed. All conflicts were resolved through discussion and consensus or by consulting the senior author. Samueli Institute developed a rulebook to ensure objectivity in scoring and reliability between reviewers to improve the often subjective assessments in quality criteria scoring in systematic reviews. As detailed above and in Table 1, the individual criteria were “weighted” to account for the omission of criteria that did not apply to this body of literature.

### 2.4. Results

**Figure 1:**
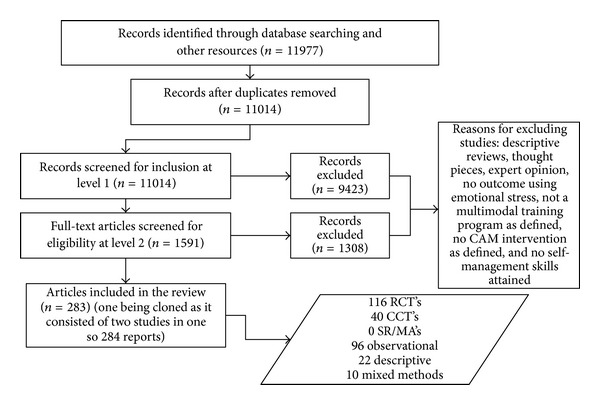
Flowchart of study selection process.

The initial search from the full systematic review yielded 11,977 citations from database inception through February 2009, of which 284 reports were deemed suitable to be included, with 116 being RCT study designs. See Figure 1 for the flow diagram of studies throughout the review phases. This current report includes only those that were RCT's and were scored as high quality and statistically significant results. Subsequent planned reports will comment on other study designs available.

### 2.5. Types of Programs

The 116 RCT studies from the full systematic review were categorized into the following types of programs: those that have been previously characterized/named (56 total): Mindfulness-Based Stress Reduction (MBSR), Cognitive Behavioral Stress Management (CBSM), Autogenic Training (AT), Relaxation Response Training (RRT), Stress Inoculation Training (SIT), Anxiety Management Training (AMT), and Coping Skills Training (CST); and those that have not been previously characterized/named (60 total): yoga and similar meditation-based modalities (including programs that incorporated yoga-type techniques as the primary intervention) and relaxation and other similar mind-body skills (including programs that used any relaxation technique, breathing, guided imagery, self-hypnosis, and/or Cognitive Behavioral Therapy (CBT) as the primary intervention).  Table 2 displays the number of RCT studies, categorized by name of program, quality rating (SIGN 50 score), and significance level showing between-group differences on stress-related outcome results. Of note, very few of the high-quality studies reported negative results. None of the studies that used CST, AMT, or SIT as an intervention fit the criteria of high quality; therefore, these will not be reported on further in this report but will be described in subsequent publications. Detailed descriptions of each of the 34 studies that were of high quality and yielded statistically significant results between groups are displayed in Table 3 and described below. Because the unnamed programs' content and heterogeneity varied across studies, the authors provide a full description of the program incorporated in each study in Table 3(b).

### 2.6. Descriptive Overview of Included High-Quality Programs

#### 2.6.1. Mindfulness-Based Stress Reduction (MBSR)

Mindfulness-Based Stress Reduction was developed approximately 30 years ago by Dr. Jon Kabat-Zinn and now has evolved into a structured group program (http://www.umassmed.edu/cfm/stress/index.aspx). It uses meditation as a tool to cultivate conscious awareness in a nonjudgmental and accepting manner. MBSR has been used to help individuals with stress, chronic pain, anxiety, sleep, and headache, among others [18, 19]. The MBSR course schedule generally consists of eight weekly classes and one day-long retreat, including guided instruction on mindfulness meditation practices, gentle stretching and mindful yoga, group dialogue and discussions aimed at enhancing awareness in everyday life, individually tailored instruction, daily home assignments, and home practice CDs.

#### 2.6.2. Cognitive Behavioral Stress Management (CBSM)

Cognitive Behavioral Stress Management is a multimodal program adapted from a variety of meditation and cognitive behavioral strategies and has been used for more than 20 years by a variety of groups. CBSM has been used to help individuals with coping, quality of life, psychological well-being, PTSD, and HIV-related stressors [20]. CBSM is generally a ten-week group-based program that combines relaxation, imagery, and deep breathing, along with cognitive behavior therapy, which is designed to help reduce bodily tension, intrusive stressful thoughts, and negative moods and improve interpersonal communication skills [21].

#### 2.6.3. Autogenic Training (AT)

Autogenic Training was developed by the German psychiatrist Johannes Schultz in 1932. The goal of AT is to achieve deep relaxation and reduce stress by teaching the body to respond to verbal commands “telling” it to relax and control breathing, blood pressure, heartbeat, and body temperature [22]. It includes standardized self-suggestion exercises to make the body feel warm, heavy, and relaxed [23–26].

#### 2.6.4. Relaxation Response Training (RRT)

Relaxation Response Training is a stress-management approach first published in 1974 by the cardiologist Benson et al. [27]. Benson found that meditation was related to general reversal of the sympathetic activation that produces the “stress response” (i.e., decreased oxygen consumption, carbon dioxide production, respiratory rate, and minute ventilation) [28]. RRT was originally based on transcendental meditation but differentiated into its own technique using the following four elements to elicit the relaxation response: (1) a mental device (e.g., a sound, word, or phrase repeated silently or audibly to free one's self from logical, externally oriented thought); (2) a passive attitude (e.g., not worrying about how well one is performing the technique); (3) a decreased muscle tonus (e.g., comfortable, relaxed posture); and (4) a quiet environment with minimal environmental stimuli (e.g., a place of worship) [29].

#### 2.6.5. Yoga + Similar Meditation-Based Skills

This miscellaneous category included studies that were not “named programs” and did not fit into any of the previously characterized categories and so were compiled together as a single, distinct category. These interventions met the inclusion criteria as programs that incorporated at least one yoga-based or meditation-based element as primary intervention. See Table 3(b) for a more complete description of each of these programs.

#### 2.6.6. Relaxation + Similar Mind-Body Skills

Another miscellaneous category also included studies of programs that did not fit into any of the previously named categories. These incorporated at least one relaxation technique (such as progressive muscle relaxation) combined with other modalities such as breathing, guided imagery, and/or Cognitive-Behavioral Therapy (CBT). While all of these studies had in common that they integrated at least one relaxation technique, for ease of discussion, the authors have grouped them into three subcategories: those that were based on a CBT model, those that were characterized as “Stress-Management Training Programs,” and those that combined relaxation with either guided imagery or breathing techniques. Cognitive-behavioral therapy is a well-established and effective psychotherapy approach for conditions such as anxiety and depression [62, 63], which assist individuals to recognize distorted thoughts, devise strategies to reframe them, and change resultant reactions and behaviors (www.nacbt.org). An example of a CBT program that incorporates a relaxation component includes sessions on understanding the nature of stress and stress reactions, breathing and/or relaxation techniques, and cognitive restructuring techniques (i.e., self-talk skills to use in stressful situations) [51].

Stress-Management Training Programs (SMTPs) incorporate a number of skills and techniques to assist individuals to attenuate their physiological and psychological reactivity to stressful situations, including problem-solving, assertiveness training, and coping skills with various relaxation techniques, such as breathing and Progressive Muscle Relaxation (PMR). In this review, a program was tagged as “SMTP” only if the intervention was described using this term.

Guided imagery (GI) is a relaxation technique that focuses on and directs the imagination to produce therapeutic change (www.healthjourneys.com) and can be administered by a trained practitioner leading an individual or group session or delivered as a recording. GI frequently includes suggestions for breathing and relaxation, followed by a purposeful directing of the imaginal mind to recreate a relaxing scene with sensory recruitment to enhance a multisensory experience. See Table 3(b) for a more complete description of these programs.

### 2.7. Results of the High-Quality Studies Included

Of the 13 MBSR studies included, seven high-quality reports (3++ and 4+) were found to produce statistically significant effects on outcomes of distress in 63 rheumatoid arthritis patients [30]; perceived stress in 47 undergraduate students [33]; anxiety and perceived stress in 109 cancer patients [34]; anxiety and distress in 78 premedical students [32]; anxiety in 20 heart disease patients [31]; distress in 104 premedical students [25]; and distress and perceived stress in 103 volunteers with high levels of perceived stress [26].

Of the 14 CBSM studies included, eight high-quality reports (2++ and 6+) were found to demonstrate statistically significant effects on outcomes of coping in 387 HIV patients [35]; everyday life stress in 198 heart disease patients [36]; anxiety in 104 HIV patients [37]; anxiety in 37 third semester economic students [38]; perceived stress in 48 university students [39]; coping and relaxation in 199 breast cancer patients [40]; anxiety in 199 breast cancer patients [41]; and coping in 52 HIV patients [42].

Of the 10 AT studies included, three high-quality reports (3+) were found to yield statistically significant effects on outcomes of anxiety in 93 nursing students with anxiety [44]; anxiety in 100 acute myocardial infarction or coronary artery bypass surgery patients [43]; and distress in 56 patients with chronic tension headache [45]. This final study included a combination program consisting of AT and self-hypnosis [45].

Of the five studies on RRT included, two high-quality reports (2+) were found to have statistically significant outcomes of psychological distress in 128 healthy undergraduate and graduate students (a program involving RRT and CBT training) [46] and distress in 80 patients with psychosomatic complaints [47].

Included in the yoga or meditation-based category were three high-quality reports (3+) that were statistically significant on measured outcomes of the study's reports. These three studies consisted of yoga, meditation and relaxation, breathing or imagery, or a combination of all, which measured perceived stress in 259 participants who had experienced a hurtful interpersonal experience from which they still felt negative emotional consequence [48] and two studies on women with breast cancer that were statistically significant on outcomes of trait anxiety stress plus state anxiety according to STAI (*n* = 34) [49] and an integrated yoga program (*n* = 98) [50].

In this relaxation-based category, 35 of 45 studies reported statistically significant differences in stress-related outcomes; 11 (24%) were classified as high quality (++ or +). Four studies examined CBT-based programs that incorporated relaxation techniques. In a study of 108 patients with severe mental illness and PTSD, an eight-session CBT program with breathing techniques resulted in significant improvements in trauma-related cognitions, anxiety, and PTSD symptoms [51]. In another study of 31 chronic schizophrenic inpatients, a 12-hour CBT program with breathing exercises resulted in significant reductions in work-related stress [53]. In a third study, a 60-hour CBT program with qi gong (a meditative breathing technique) and relaxation exercises resulted in significantly less burnout in 75 individuals with stress-related conditions [52]. In the fourth study, 85 pediatric headache patients who completed a 12-hour CBT program incorporating progressive muscle relaxation had significant improvements in coping with stress [54]. Three studies characterized their intervention as an SMTP plus relaxation techniques. In one study of 81 US Army employees stationed at the Pentagon, an SMTP that combined multiple relaxation techniques resulted in significant reductions in distress but not anxiety [55]. A second study which implemented a 16-hour SMTP with PMR in 36 parents of children with severe physical disabilities resulted in significant reductions in both state and trait anxiety [56]. A third study using a 20-hour SMTP program with PMR resulted in decreases in distress and trait anxiety in 155 police, hospital, and school employees [57].

Four-high quality studies implemented GI or breathing exercises with other relaxation techniques, and in each case the results were mixed: some stress-related outcome measures showed statistically significant differences, while others did not. In a study of 134 ischemic heart disease patients, a 24-hour program combining GI with Coping Skills Training and PMR resulted in significant reductions in distress but not in anxiety [58]. A second study implemented a nine-hour program of GI with Coping Skills Training and PMR in 50 breast cancer patients and found a decrease in anxiety as measured by the Profile of Mood States (POMS) but not by the Hamilton Anxiety and Depression Scale (HADS) [60]. The third study used three hours of GI with breathing and relaxation exercises in 161 breast cancer patients undergoing radiotherapy; while tension scores on the POMS lessened significantly, the Leeds anxiety differences were nonsignificant [59]. The fourth study that used relaxation, breathing, and desensitization techniques in highly anxious psychology students produced statistically significant reductions in test-taking anxiety [61].

### 2.8. Resource Requirements of Named Multimodal Programs

**Figure 2:**
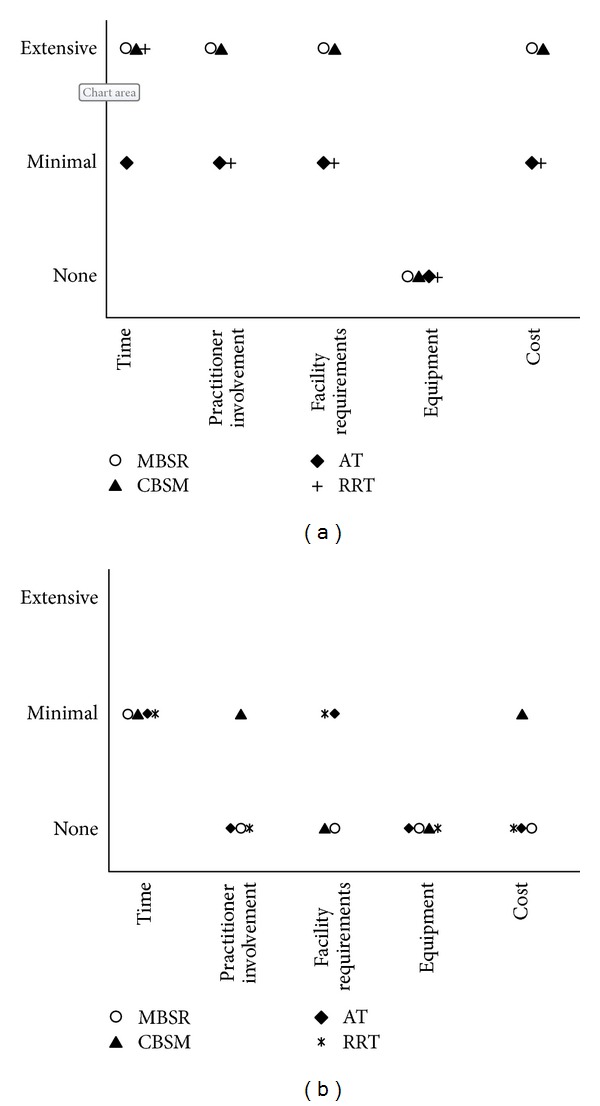
(a) Training requirements and (b) self-practice requirements.

In this section, the authors provide a subjective assessment of the resource requirements for these programs, based on the descriptive data collected: information describing the amount of time required for individual and practitioner or trainer involvement, facility and equipment needed, and estimated cost ranges. Because the unnamed programs were heterogeneous with regard to time for training and content involved in each session, the authors elected not to report on resource requirements for these. In determining what resources would potentially be required during the training phase for the named programs (see Figure 2(a)), the authors considered this as the period of time when a program was initially instituted and would require a trainer or instructor to teach self-management skills to participants. Since data was collected on the “dose” of the program training (i.e., the actual number of hours per week × number of weeks in which the program was delivered), the authors then categorized the amount of training time needed as minimal (less than 10 hours on average) or extensive (greater than 10 hours). Based on this information, the authors then estimated the amount of practitioner or trainer involvement required to teach these skills. Programs like MBSR and CBSM require substantially more specialized training of and sustained practitioner involvement, compared to interventions like AT and RRT which can be more quickly learned by participants. Using the descriptions of the intervention extracted from the data, the authors then codified facility requirements (i.e., an estimate of how much space is needed to learn the techniques), whether any equipment is necessary to learn the skills, and costs associated with the training (based on internet searches of the described programs). The authors present a similar assessment for the self-practice requirements (i.e., once the individual is fully trained and able to practice on his/her own) in Figure 2(b). Compared to conventional therapies, the resource requirements for both training and self-practice are all overall likely minimal. Once fully trained, service members should be able to practice these skills easily in any setting, with minimal time required, no equipment necessary, and at virtually no additional cost. While the main focus of this review was to report on the effectiveness of these multimodal programs in impacting emotional stress, the authors have additionally provided information about estimated resource requirements for military leadership and program managers in order to guide their decision making about the feasibility of integrating such programs into military settings. Whether these programs could be implemented “as is” or if they would need to be modified or adapted is not an assessment the authors have made, as only those in decision-making positions are able to definitively decide such feasibility issues.

## 3. Discussion

The programs described in this report have potential benefits for service members and their families. Since they primarily involve self-management skills, they can become self-empowering to the individual and can be used in any environment, with minimal time needed. This may be especially helpful to the population of individuals that are likely to refuse, delay, or feel stigmatized by conventional therapies. They are cost-effective strategies to prevent or manage stressors. Since they are multimodal, they may offer greater appeal than single-modality programs. There are very few to no adverse effects from these self-management skills when properly learned and practiced.

Although there are reviews in the literature to describe relevant programs that address specific issues (PTSD, resilience) in service member populations [64, 65], this comprehensive systematic review globally reviewed the literature on biopsychosocial multimodal programs, extracted the ones with high methodological quality and statistically significant reductions in stress (and similar keywords), and presented these results with estimated resource requirements. Because of this comprehensive approach, promising programs that have a strong evidence base, most of which were not evaluated in military populations, were able to be identified. This information is important for learning approaches that could be applied in these populations, especially since there is so little research on these topics in military populations. This data could potentially aid military leaders who are looking for evidence-based programs to reduce psychological stress and help guide their decision making about implementing these programs as described, or tailoring the needs of service members.

A fundamental problem associated with initial efforts to launch effective mind-body programs in practice is the limited evidence base to guide program choice. While evidence-based approaches may be desirable, such evidence is scarce. Even if evidence is available, the basic steps of program planning may lead conscientious planners to programs that have not been evaluated for their effectiveness. With this paper, the authors hope to stimulate thinking about translating this best evidence synthesis into practice, in order to make headway into the prevention and treatment of stress-related illness. The message to service members and their family members who are struggling with stress-related conditions is that they can change the way their body and mind react to stress by changing their thoughts, emotions, and behaviors. To the leadership, that is, understandably wary of making decisions without the proper evidentiary support, the authors offer the results of this comprehensive systematic review, demonstrating some promising directions, preliminary evidence of effectiveness for stress-related outcomes across *all* populations, and basic characterizations and descriptions of some of the self-care, skills training programs. The goals were to demystify them and to illustrate that many of them involve minimal cost and training time.

The US military success at shifting the combat focus from response to IED attacks in theater to better IED detection and armor to prevent injuries, termed “left of boom” [66] can serve as a powerful model for the mitigation of combat stress-related issues. A similar commitment to changing the paradigm from treatment of combat-related PTSD after it has been diagnosed to empowerment of troops and their families to take control over their physiologic and psychological responses to stress through skills training would represent a true shift to “left of PTSD” [67, 68].

### 3.1. Limitations of the Review

The authors of this comprehensive systematic review were only interested in assessing and reporting on the stress-related outcomes reported in the articles; whether they were primary or secondary outcomes was irrelevant to the review. Because of this, the authors did not paint the whole picture of each study captured and may have missed important elements of the original authors' intent. It was chosen to capture only those studies that reported on stress using the following terms: *stress*, *anxiety*, *posttraumatic stress disorder*, *coping*, *resilience*, *hardiness*, *burnout*, *distress*, or *relaxation*. The authors derived these terms by assessing the literature and consulting with subject matter experts. The authors acknowledge that this is most likely not a comprehensive and exhaustive list of all emotional stress-related terms, and the search may have missed pertinent studies that would have fit the inclusion criteria using other similarly related terms for stress.

Another limitation is that this review only included multimodal programs. The authors felt that these programs would include the dimensions of the BPS model and would have broader appeal by not focusing all efforts on one technique (e.g., yoga). As a result, any studies involving a single modality (i.e., only yoga, meditation, relaxation, or imagery) were excluded at the screening phase. The authors and colleagues are currently assessing the literature of single-modal mind-body approaches in a more focused population relating to the military.

The authors only included those studies that have been published in the English language. While some systematic reviews consider the inclusion of only English-language studies as a limitation; doing so does not seriously compromise the outcome or implication for the majority of interventions and claims [69]. There has been some debate over this in the literature. The amount of effort and expense to include studies that have not been translated into the English language is a challenge to methodologists since the translator needs to be proficient in scientific language, able to understand the systematic review methodology, and be involved from the protocol development phase to clearly understand how to accurately code each review. The majority of systematic reviews, because of this challenge, only include the literature that is available in the English language.

The authors excluded all biofeedback studies during the review phase because it was decided that these were not truly self-management techniques; one would have to rely on the device during practice. Other programs, such as those learned through the internet or books, were excluded on this basis as well.

In this report, the authors have only described those studies that were of high quality and which reported statistically significant results between groups in controlled trial designs because of the interest in sharing only those that the authors felt confident in the estimate of the effect compared to a control group. Thus, excluded from this report were those programs that showed only within-group differences. Finally, quality assessment was based solely on internal validity criteria (the likelihood that the observed effects are due to bias) and did not take into account external validity (the likelihood that observed effects would occur outside the setting, i.e., generalizability), which is not usually assessed when evaluating quality in systematic review. Had the authors assessed external validity, the number of low-quality studies may have been decreased, allowing more studies to be shared. Future studies in the field should evaluate not only bias but also generalizability when assessing quality criteria.

## 4. Conclusion

The objective of this paper was to provide a descriptive overview and quantitative synthesis of information on multimodal programs that might be used for the self-management of emotional stress in our military communities and to consider this body of research as a guide to next steps in the research on implementation in military populations. MBSR, CBSM, AT, RRT, yoga plus similar meditation-based skills, and relaxation practices are the types of approaches emerging in the literature as the most promising for their benefits and ease of implementation in different settings. Implementing these identified training programs into military settings appears highly feasible, considering that resource requirements are minimal. 

## Figures and Tables

**Box 1:**
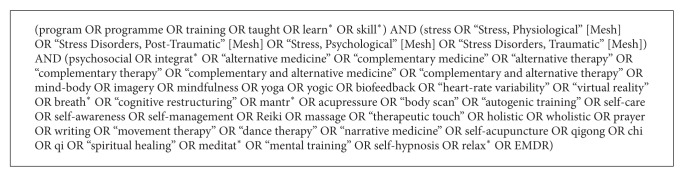
Search terms used according to MeSH strategy.

**Table:** (a) Section  1: internal validity*

Item	Description
1.1	The study addresses appropriate and clearly focused question.
1.2	The assignment of subjects to treatment groups is randomized.
1.5	The treatment and control groups are similar at the start of the trial.
1.7	All relevant outcomes are measured in a standard, valid and reliable way.
1.8	What percentage of subjects in each treatment arm dropped out before the study was completed?
1.9	All subjects are analyzed in the groups to which they were randomly allocated (intention to treat analysis).
1.10	Where the study is carried out at more than one site, results are comparable for all sites.

Each item in Section  1 is to be evaluated using these criteria: well covered; adequately addressed; poorly addressed; and not applicable (NA) only for question 1.10.

*Note that 1.3, 1.4, and 1.6 SIGN criteria were omitted from our modified version of the SIGN as they did not apply to our research question/population: as there were wide differences in the types of programs assessed. Note that all criteria were weighted according to a revised SIGN quality score as reflected below consistently.

**Table:** (b) Section  2: overall assessment

	How well was the study done to minimize bias? How valid is the study? Score options: ++, +, and − based on the following (modifications to SIGN criteria in italics).
++	All or most of the criteria have been fulfilled. Where they have not been fulfilled, the conclusions of the study are thought “very unlikely” to alter. An article receives this score if there are 0 criteria scored as poorly addressed.
+	Some of the criteria have been fulfilled. Those criteria that have not been fulfilled or not adequately described are thought “unlikely” to alter the conclusions. An article receives this score if 1-2 criteria are scored poorly addressed.
−	Few or no criteria fulfilled. The conclusions of the study are thought “likely or very likely” to alter. An article receives this score if more than 2 criteria are scored as poorly addressed.

SIGN 50 network: a guideline developer's handbook http://www.sign.ac.uk/guidelines/fulltext/50/checklist2.html.

**Table 2:** Randomized controlled trials (*n* = 116) by SIGN score and significance level*.

SIGN quality rating	**++ **	**+ **	−	++	+	−	Total
Significance level*	*P* < 0.05			*P* > 0.05		
Mindfulness-Based Stress Reduction (MBSR)	**3**	**4**	3	0	2	1	13
Cognitive Behavioral Stress Management (CBSM)	**2**	**6**	5	0	1	0	14
Autogenic Training (AT)	**0**	**3**	4	1	1	1	10
Relaxation Response Training (RRT)	**0**	**2**	1	0	1	1	5
Yoga + Similar Meditation Techniques	**0**	**3**	8	1	2	1	15
Relaxation + Similar Mind-Body Techniques			24	0	4	6	45
(1) Cognitive-Behavioral Therapy-Based Programs	**1**	**3**					
(2) Stress-Management Training Programs (SMTP)	**0**	**3**					
(3) Guided Imagery and/or Relaxation, and Breathing Techniques	**1**	**3**					
Coping Skills Training (CST)	**0**	**0**	3	0	0	0	3
Anxiety Management Training (AMT)	**0**	**0**	4	0	0	0	4
Stress Inoculation Training (SIT)	**0**	**0**	7	0	0	0	7
**Totals**	**7**	**27**	59	2	11	10	116

*On stress-related outcomes with keywords: stress, anxiety, post-traumatic stress disorder, coping, resilience, hardiness, burnout, distress, or relaxation.

**Table:** (a) Named programs

Citation	Population	Intervention/control	Number assigned (dropout %)	Total dose	Stress outcomes (between group differences)	Conclusions	Quality
Mindfulness-Based Stress Reduction (MBSR)							

Pradhan et al., 2007 [30]	63 male and female rheumatoid arthritis patients Mean age: 54	MBSR/WLC	T 31 (7%)/C 32 (7%) (a)	2.5 h × 8 w (20 h + one-day retreat + homework)	SCL-90 (revised): psychological distress (*F* = 4.02, 56 df, *P* = 0.04) at 6 months (d)	Significant improvement in psychological distress and 35% reduction in psychological distress among those treated	++

Tacón et al., 2003 [31]	20 women with heart disease Mean age: T 57.3, C 63.6	MBSR/WLC	T 10/C 10; dropouts: 10% in each group (b)	2 h × 8 w (16 h + homework + retreat)	STAI (state anxiety): *F* (1, 16) D 6.79, *P* < 0.01 (d)	Significant differences between the treatment and control groups on scores of anxiety, emotional control, and reactive coping in women diagnosed with heart disease	+

Shapiro et al., 1998 [32]	78 male/female medical students experiencing stress Mean age: ND	MBSR/WLC	T 37 (3%)/C 41 (9.8%) (b)	2.5 h × 7 w (17.5 + homework)	SCL-90 (revised): psychological distress and GSI (*P* < 0.02) and STAI anxiety (*P* < 0.05) (d)	Significantly reduced self-reported state and trait anxiety and reduced reports of overall psychological distress including depression, at termination of intervention	+

Oman et al., 2008 [33]	47 undergraduate students Mean age: 18–24	MBSR condensed/Easwaran's 8-point program EPP/WLC	MBSR 16 and EPP 16/C 15; 7% total dropout (a)	1.5 h × 8 w (12 h)	PSS: (*P* < 0.05, Cohen's *d* = 0.45)	Significant larger decreases in perceived stress in the treatment group compared to control, implying that meditation-based stress management practices reduce stress among undergraduates	++

Speca et al., 2000 [34]	109 male/female cancer outpatients with various stages of disease Mean age: T 54.9, C 48.9	MBSR condensed/WLC	T 61 (13%)/C 48 (23%) (b)	1.5 h × 7 w (10.5 + homework)	POMS (anxiety): *P* < 0.001 in treatment group from time 1 to time 2 and *P* < 0.001 between the two groups; total stress score (*t* (88) 5–22.80, *P* < 0.01) (d)	Significant decreases in mood disturbance and stress symptoms in both male and female patients with a wide variety of cancer diagnoses, stages of illness, and ages	++

Jain et al., 2007 [25]	104 male/female medical students, graduate nursing students, and undergraduate students Mean age: 25	MBSR condensed (MM)/stress reduction (SR)/control group	ND; dropouts: 23%, 23 participants (6 MM, 11 SR, and 6 controls) (a)	1.5 h × 4 (6 h + homework + retreat)	BSI: distress for MM and SR versus control group (*P* < 0.05 in all cases). Effect sizes for distress were large for both meditation and relaxation (Cohen's *d* = 1.36 and .91, resp.)	Both MM and SR are effective in reducing negative psychological states and enhancing positive states of mind for students experiencing significant distress. There were no significant differences between meditation and relaxation on distress over time	+

Williams et al., 2001 [26]	103 male/female community volunteers with high perceived stress Mean age: 49.2	MBSR modified/group given educational materials on stress management and referral to community resources	T 59 (45%)/C 44 (41%) (a)	2.5 h × 8 w (20 h + one-day retreat)	DSI, SCL90-R (GSI). GSI (between group analysis): postintervention was borderline significant (*P* = 0.057) and became significant at 3-month followup (*P* = 0.049) (d)	Significant reductions in perceived stress and psychological distress found both between groups and within treatment group from pre- to postintervention	+

Cognitive Behavioral Stress Management (CBSM)							

McCain et al., 2008 [35]	387 male/female HIV + individuals Mean age: 42.2	Cognitive behavioral relaxation training (RLXN)/focused Tai chi training (TCHI)/spiritual growth group (SPRT)/WLC	Unclear (overall dropout rate 35% (a)	1.5 h × 10 w (15 h + homework)	Coping subscale of the DIS: *P* < 0.030 for emotion-focused coping; the RLXN and TCHI treatment groups showed significant total treatment effects over the control (d)	In comparison to WLC, both RLXN and TCHI groups less frequently used emotion-focused coping strategies. Generally, decreased emotion-focused coping can be considered an enhancement in coping strategies; however, there was no concurrent increase in problem-focused or appraisal-focused coping, making interpretation of this change more tenuous	++

Claesson et al., 2005 [36]	198 ischemic heart disease women Mean age: T 59, C 62	CBSM/usual care	T 101 (20.8%)/C 97 (11.3%) (a)	2 h × 20 (40 h)	ELSS: group by time interaction effect from baseline to followup *P* = 0.006 (d)	A 1-year CBSM program designed specifically for women significantly improved psychological well-being in some aspects in comparison to usual care	++

Berger et al., 2008 [37]	104 male/female HIV-infected persons Mean age: 44	CBSM/standard care	T 53 (34%)/C 51 (18%) (a)	2 h × 12 w (24 h + homework)	HADS (anxiety): changes from baseline to 12 weeks between groups (−2.4 (−4.0–−0.9)*P* = 0.003) Effect size: *d* = .52 for CBSM baseline to 12 months on HADS anxiety	CBSM training of HIV-infected persons taking cART does not improve clinical outcome but has lasting effects on quality of life and psychological well-being	+

Gaab et al., 2006 [38]	37 healthy 3rd semester economics students Mean age: ND	CBSM/control not specified	4 groups of 8–10 subjects. (CBSM groups 1 and 2: *N* = 18, control groups 3 and 4: *N* = 19); dropouts: T 28%/C 22% (b)	6 h × 2 d (12 h + homework)	MESA: between groups *F* (6, 19) = 1.30, *P* = 0.3, STAI (trait) CBSM = STAI (state) *F *((2.06/53.59) Z3.84, *P* = 0.03. Effect size: state anxiety *f* ^2^ = 0.32	CBSM prevents increases in anxiety and somatic symptoms prior to an upcoming stressor and influences the ability to exert a cortisol response corresponding to the subjective stress appraisal	+

Gaab et al., 2003 [39]	48 male students Mean age: T 24.2, C 24.5	CBSM/WLC	T 24 (unclear)/C 24 (unclear) (a)	6 h × 2 d (12 h + homework)	PSS: group by time interaction effect from baseline to posttreatment (*t*35 = 2.57, *P* < 0.02): *F* (1/46) = 5.27, *P* = 0.026, effect size *f* ^2^ = 0.11)	Short, group-based, Cognitive Behavioral Stress Management training reduces the salivary free cortisol stress response to an acute stressor in healthy male subjects with treatment group showing a reduction in the level of perceived stress posttreatment	+

Antoni et al., 2006 [40]	199 female breast cancer patients (stage III or less) Mean age: 50	CBSM and relaxation/condensed educational intervention or social support	T 92 (22%)/C 107 (19%) (b)	2 h × 10 w (20 h + homework)	MCOS: between groups changes from baseline to 10 weeks for relaxation (*P* = 0.001) and coping (*P* = 0.06). Effect size Cohen's *d* MCOS relaxation = 0.86, coping *d* = 0.04	The intervention increased confidence in being able to relax at will. There was also evidence that effects of the intervention on the various outcomes examined were mediated by change in confidence about being able to relax	+

Antoni et al., 2006 [41]	199 female nonmetastatic breast cancer patients at stage III or below and surgery within the past 8 weeks Mean age: 50	CBSM and relaxation/condensed educational intervention	T 92 (19.5%)/C 107 (22.4%) (b)	2 h × 10 w (20 h + 1-year followup)	HADS (anxiety): Group effect on slope: *z* = 2.71, *P* < 0.003; Cohen's *d* = 0.74. Affect balance scale (distress): group effect on slope: *z* = 2.48, *P* < 0.02; Cohen's *d* = 0.33. Groups differ at time 3 (*z* = 2.63, *P* < 0.01; Cohen's *d* = 0.43)	Structured, group-based cognitive behavior stress management may ameliorate cancer-related anxiety during active medical treatment for breast cancer and for 1 year following treatment	+

Lutgendorf et al., 1998 [42]	52 HIV + males Mean age: 36.75	CBSM and relaxation/WLC with one-day didactic and experiential stress management program	T 26 (19%)/C 26 (30%) (b)	135 m × 10 w (22.5 h + homework)	COPE (60-item scale) for coping: (*P* < 0.05) (d)	Significantly greater improvement in active coping than controls. Group-based CBT+ stress management significantly attenuated anxiety in HIV-positive men	+

Autogenic Training (AT)							

Trzcieniecka-Green and Steptoe, 1996 [43]	87 men and 13 women admitted to hospitals for acute myocardial infarction (WHO criteria) or coronary artery bypass surgery Mean age: <70	Autogenic Training/WLC	T 50 (ND)/C 50 (ND) (c)	10 sessions (ND)	HAD (anxiety): group by time interactions *P* < 0.01, and at followup *P* < 0.05. (d)	Significant reductions in anxiety in treatment group. Stress management training may lead to improvements in the quality of life of myocardial infarction and coronary artery bypass patients	+

Kanji et al., 2006 [44]	93 male/female nursing students Age range: 19–49	Autogenic Training/attention control using laughter therapy/time control with no treatment	T 32 (34%)/attention control 30 (20%)/time control 31 (16%) (a)	1 h × 8 w (8 h + homework)	STAI: state anxiety between treatment and time control (*P* < 0.001) between treatment group and attention control (*P* < 0.005), and between the two control groups (*P* < 0.595). Trait anxiety between the treatment and time control groups (*P* < 0.001) and between the treatment group and the attention control group (*P* < 0.084) (d)	Autogenic Training is significantly more effective in reduction of state and trait anxieties than in both other groups immediately after treatment	+

Spinhoven et al., 1992 [45]	56 male/female patients with tension headache Mean age: 36	Autogenic Training and self-hypnosis (SH)/WLC	ND (b)	45 m × 4 (3 h + homework + 3 boosters)	SCL 90: psychological distress *P* < 0.01 at posttreatment and level of psychological distress in contrast to the waiting-list period (*P* < 0.05). Follow-up measurements indicated that therapeutic improvement was maintained (*P* < 0.05). CSQ: *P* = 0.003 at posttreatment (d)	Patients treated with AT or SH training achieved moderate reductions in psychological distress and showed statistically significant reductions in distress compared to WLC	+

Relaxation Response Training (RRT)							

Deckro et al., 2002 [46]	51 men and 77 women students Mean age: 24	Relaxation Response and CBT Training/WLC	T 63 (13%)/C 65 (16%) (b)	1.5 h × 6 (9 h + homework)	GSI (SCL-90-R) (psychological distress); between group analysis from baseline to postintervention: (*P* = 0.018). PSS: within group analysis: pre-post scores for intervention versus control group (*P* = 0.008); STAI state anxiety (*P* = 0.001) (d)	A 6-week RR and CBT training program significantly reduce self-reported psychological distress, anxiety, and the perception of stress	+

Hellman et al., 1990 [47]	80 male/female patients with psychosomatic complaints Mean age: 37	Relaxation Response Training/stress management information group	Ways to wellness 28/mind/body program 27/stress management info. group 25; 11% total dropouts (b)	WTW and MBP: 1.5 h × 6 w (9 h + homework), SMG: 1.5 h × 2 (3 h)	B-POMS: between groups analysis-psychological distress for both WTW and MB groups (*t* = 4.02, *P* < 0.01), a decline that was significantly greater than that for the information group (*P* < 0.05) (d)	At the 6-month followup, patients in the behavioral medicine groups showed significantly greater reductions in visits to the HMO and in discomfort from physical and psychological symptoms than did the patients in the information group	+

**Table:** (b) Unnamed programs

Citation	Population	Intervention/control	Description of program	Number assigned (dropout %)	Total dose	Stress outcomes (between group differences)	Conclusions	Quality
Yoga + Similar Meditation-Based Skills								

Harris et al., 2006 [48]	259 male/female participants who had experienced a hurtful interpersonal experience from which they still felt negative emotional consequences Mean age: 41.8	Meditation and imagery/no treatment control	Combination of cognitive restructuring positive and negative visualizations and heart-focused meditation techniques. Time was devoted to education about the negative health consequences of grudge-holding and unforgiveness, cognitive restructuring, and meditations/relaxation exercises. Exercises used in the training were principally tailored to instill and cultivate a more relaxed state, to reduce arousal during the recollection of interpersonal grievances, and to improve participants' ability to regulate emotions by consciously shifting attention between negative and more neutral or positive thinking and feeling states	T 134 (14%)/C 125 (18%) (b)	1.5 h × 6 w (9 h)	PSS perceived stress (*P* < 0.001). Effect size: Cohen's *D* for PSS 0.66 at posttest and 0.54 at followup	Significant treatment effects were found for forgiveness self-efficacy, forgiveness generalized to new situations, and perceived stress	+

Nunes et al., 2007 [49]	34 female breast cancer patients Mean age: T 54.2, C 50.07	Meditation, relaxation, breathing, and imagery: relaxation and visual therapy (RVT)/no intervention	Relaxation and visualization therapy (RVT) intervention includes a relaxation period (20 min), in which the subject is induced to mentally create an image of the desired objective or result, including progressive muscle relaxation, guided imagery, meditation, and deep breathing. Subjects were guided to create a mental image in which their tumor is attacked by their immune system and then to visualize the breast completely healed	T 20/C 14 (0%) (b)	0.5 h × 24 (12 h + homework)	ISSL, STAI: within groups (pre- versus postexperimental group): ISSL Q1 *P* < 0.01, Q2 *P* < 0.05, Q3 *P* < 0.001. STAI (state) *P* < 0.05, trait *P* < 0.001. The psychological scores did not change over time in the control group (all *P* > 0.05). Effect sizes: SSL Q1 .72 ISSL Q2 .64 ISSL Q3 .70 STAI (State) .52 STAI (Trait).79	RVT is effective for reducing stress, anxiety, and depression scores and may improve the quality of life of cancer patients undergoing radiotherapy	+

Raghavendra et al., 2007 [50]	98 female breast cancer outpatients Mean age: ND	Yoga, breathing/psychodynamic supportive-expressive therapy with coping preparation	Yoga intervention consisted of a set of asanas (postures done with awareness), breathing exercises, pranayama (voluntarily regulated nostril breathing), meditation, and yogic relaxation techniques with imagery. These practices were based on principles of attention diversion, mindful awareness, and relaxation to cope with day-to-day stressful experiences. The first session consisted of yogic relaxation, meditation using breath awareness, and impulses of touch emanating from palms and fingers or chanting a mantra from a Vedic text for 30 min. Subjects in the yoga group were provided with audio and video cassettes of the yoga modules for home practice; these home sessions started with a few easy yoga postures, breathing exercises and pranayama (voluntarily regulated nostril breathing), and yogic relaxation	T 28/C 34l (37%) (b)	YR 30 m (.5 h + homework) Counseling 1 h; control .5 h	STAI state anxiety score: between groups analysis *P* < 0.001. Subjective questionnaires: number of distressful symptoms *P* = 0.002; symptom distress: *P* < 0.002 (d)	There was a significant decrease in reactive anxiety states, depression, number of treatment-related distressful symptoms, severity of symptoms and distress experienced, and improvement in quality of life during chemotherapy in the yoga group as compared with control	+

Relaxation + Similar Mind-Body Skills								

Mueser et al., 2008 [51]	108 severe mental illness male/female patients Mean age: 44.21	CBT and breathing/TAU with supportive counseling as needed	CBT program for PTSD included 8 modules: introduction, crisis plan review, psychoeducation (symptoms of PTSD), breathing retraining, psycho-education (associated symptoms of PTSD), cognitive restructuring (common styles of thinking), cognitive restructuring II (5 steps of cognitive restructuring), generalization training, and termination	CBT program 54 (20%)/treatment as usual (TAU) program 54 (0%) (a)	ND	PTCI, BAI, CAPS: between groups analysis CBT versus TAU (baseline versus postintervention): PTCI *P* < 0.001; BAI *P* < 0.03, CAPS Dx *P* = 0.63, CAPS Dx (>65) *P* = 0.02, CAPS Dx (<65) *P* = 0.18. Post hoc analysis: subset with severe PTSD (CAPS > 65). Effect sizes for both CAPS-total increased, from .45 to .59 and in CAPS-diagnosis from .27 to .40. Subset with mild-moderate PTSD (CAPS < 65) The effect sizes decreased to .12 and .10, respectively	Findings suggest that clients with severe mental illness and PTSD can benefit from CBT and breathing, despite severe symptoms, suicidal thinking, psychosis, and vulnerability to hospitalizations	++

Heiden et al., 2007 [52]	75 male/female patients on sick leave for at least 50% of the time for stress-related diagnoses Mean age: 44	CBT and relaxation techniques/physical activity/usual care	Cognitive intervention focused on education, qigong and relaxation techniques, coping skills, and stress management exercises. Participants in the physical activity group were offered exercise sessions. Participants chose an exercise (e.g., strength training, swimming, aerobics, or walking) in consultation with the group leader. During the intervention, each participant kept a diary of their physical exercise	CBT 28 (28%)/phys. 23 (4.3%)/control 24 (8.3%) (a)	3 h × 2 × 10 w (60 h + homework)	BQ: between groups (*F* (2, 61) = 3.9, *P* = 0.024). By 6 months the differences were not significant *P* = 0.062 (d)	CBT group reduced their burn-out ratings compared with the control group by the end of the intervention. At followup, these differences faded	+

Lee et al., 2006 [53]	31 male/female patients with chronic schizophrenia who engaged in level 4 or 5 of the center's part time paid job program Mean age: 34.9	CBT and breathing/WLC	The work-related stress management program included short lectures on the influences of stress on cognition, emotion, and behavior; instruction in the techniques of handling negative emotions and stress (e.g., deep breathing, strut (walking proudly), and exercise) and emotional intelligence. Several sessions were devoted to communication, skills training, assertiveness training, and problem-solving skills training. Finally, methods dealing with work-related crises were presented and practiced	31 total Only 2 dropout total (b)	1 h × 12 w (12 h + homework)	WSQP: between groups analysis from first to second testing period (12 weeks): total WSQP stress score *P* = 0.0039. Pooling data from both 12-week treatment periods, treatment effect for the change in total WSQP scores (*P* = 0.0034). Effect size: *r* = 0.49	Work-related stress management program had large short-term positive effects on patients' perceived work-related stress. These findings support providing this type of program to employed patients with schizophrenia	+

Kroener-Herwig and Denecke, 2002 [54]	85 out of 175 who met the inclusion criteria, male/female pediatric headache patients Mean age: 12.1	CBT and relaxation (TG)/self-help (SH) control group following the same program except that treatment done through the use of a manual/WLC	Main topics TG: session 1 is an introduction to the training as well as education about headache. Session 2 dedicated to the acquisition of progressive relaxation techniques. Session 3 introduced the perception of stress symptoms, the role of stress regarding headache and how to cope with stress. Session 4 introduced the children to the significance of dysfunctional and functional cognitions regarding stress and headache. Session 5 explained the role of attention on pain experience and introduced positive imagery as means to distract attention from pain and attain a relaxed state. In Session 6, self assertive behavior was the main topic. Session 7 offered a model for general problem solving. Session 8 gave a summary of all skills.	TG 30/SH 35/WLC 20 Dropout: 12% (unclear as to which groups) (b)	1.5 h × 8 w (12 h)	The “coping with stress” subscale of the stress questionnaire: TG and SH compared to WLC overall (*P* = 0.032) (d)	The efficiencies of the two training formats are nearly identical. Both groups significantly reduced stress as compared to the WLC. The group format, because of its better acceptance, is recommended for practical use	+

Pruitt, 1992 [55]	81 male/female US army employees Age range: 21–65	SMTP with relaxation/control group with delayed class attendance at the end of testing period	The stress management course in the “Fit to Win” program consisted of strategies involving stress awareness and principles of home management, environmental modification, and assertiveness, as well as multiple methods of relaxation. An audio cassette of relaxation strategies was available for home practice	T 31/C 33; dropouts: ND (b)	ND	STAI: pretest to posttest between groups *F* (1, 61) = 1.32 *P* = 0.254 SCL-90: pretest to posttest between groups *F* (1, 62) = 5.21 *P* = 0.026. (d)	There was no statistically significant difference between groups for state anxiety. The lack of significance is primarily due to improvements in the control group members also participating in the overall wellness program. There was a significant overall improvement for the combined groups in relation to all four variables (stress-related physical symptoms, perception of anxiety, and systolic and diastolic blood pressure). There is benefit to this program with overall low cost	+

Singer et al., 1988 [56]	36 male/female parents of children with severe handicaps Mean age: ND	SMTP with relaxation/control	Lectures, demonstrations, and discussion focused on self-monitoring of stress and physiological reactions to stress, muscle relaxation, and restructuring/modifying cognitive distortions related to stress	T 18/C 18; dropouts: ND (b)	2 h ×8 w (16 h)	STAI: analysis of covariance (controlling preintervention scores) state and trait anxiety *F* (1, 34) = 5.98, *P* = 0.02. (d)	The treatment group improved significantly on measures of depression and anxiety	+

de Jong and Emmelkamp, 2000 [57]	155 males/females recruited through employers Mean age: 38	SMTP with relaxation (different groups of workers)/assessment of only control group	The SMT program taught participants a variety of active coping strategies covering the following elements: (a) progressive muscle relaxation, (b) problem-solving training, (c) assertiveness skills training, and (d) raising awareness of individual stressors, stress reactions, coping style or styles, and (un)healthy lifestyle. At the outset of each session, an outlined agenda was provided. Agendas included theoretical lectures, exercises (i.e., relaxation and problem-solving exercises and behavioral role play with other group members), and homework assignments	SMTpsy 53 (11%)/SMT para 51 (14%)/controls 51 (20%) (b)	2.5 h × 8 w (20 h + homework)	GHQ for general distress and STAI (trait): difference between the intervention and control for both measures *P* < 0.05. (d)	Results show favorable effects of the SMT program both in the short term and at 6-month followup. Results showed no serious differences in effectiveness between trainers. It is argued that, to be effective, the SMT program does not necessarily have to be given by clinical psychologists only but may instead be given by individuals from other professional orientations	+

Blumenthal et al., 2005 [58]	134 ischemic heart disease (IHD) patients Mean age: 63	Relaxation and imagery(SM)/ exercise only/usual care	3 key components to stress management (SM) training: education in which participants were provided information about IHD and myocardial ischemia, structure and function of the heart, traditional risk factors, and emotional stress. Second, patients underwent skills training, involving instruction in specific skills to reduce the affective, behavioral, cognitive, and physiological components of stress. Therapeutic techniques included graded task assignments, monitoring irrational automatic thoughts, and generating alternative interpretations of situations or unrealistic thought patterns. Patients instructed in progressive muscle relaxation and imagery techniques, along with training in assertiveness, problem solving, and time management. Role-playing also was used. Third, group interaction and social support were encouraged	SM 44 (5%)/exercise 48 (8%)/usual care 42 (9.5%) (a)	1.5 h × 16 w (24 h)	STAI general anxiety: *P* = 0.22 for exercise and SM versus usual care after treatment and the 24-item GHQ to assess psychiatric symptoms and general distress *P* = 0.02 for exercise and stress management versus usual care (d)	For patients with stable IHD, exercise and stress management training reduced emotional distress and improved markers of cardiovascular risk more than usual medical care alone	++

Bridge et al., 1988 [59]	161 females with breast cancer stage I or II after first session of six-week course of radiotherapy Mean ages: R&I 53, R 51, control 54	Relaxation, breathing, and imagery/relaxation/ control group	Both treatment groups (relaxation and relaxation plus imagery) were taught a relaxation technique which by a process of direct concentration focuses sensory awareness on a series of individual muscle groups. These patients were also given instructions for diaphragmatic breathing, which slows respiration, induces a calmer state, and reduces tension. In addition to the breathing and relaxation, each patient in the relaxation plus imagery group was taught to imagine a peaceful scene of her own choice as a means of enhancing the relaxation.	Unclear; 13% total dropout b	.5 h × 6 w (3 h + homework)	The item “relaxed” is part of the subscale for tension in the POMS: *P* = 0.025 The Leeds general scales for anxiety and depression showed no significant changes over the six weeks of treatment (d)	At the end of the study period the women trained in relaxation plus imagery were more relaxed than those trained in relaxation only, who in turn were more relaxed than the controls. Patients with early breast cancer benefit from relaxation training	+

Fukui et al., 2000 [60]	50 female breast cancer patients Mean age: T 52.6, C 54.3	Relaxation and imagery/WLC	The model consisted of these components: (1) health education; (2) Coping Skills Training; (3) stress management; and (4) psychosocial support. In the health education component, medical and psychologic information specific to breast carcinoma was presented. In the coping skills component, the patients were taught to utilize the active-cognitive and active-behavioral coping methods when they encountered specific problems related to having cancer. In the stress management component, they were taught relaxation exercises, including progressive muscle relaxation (PMR) followed by guided imagery (GI). Psychologic support was offered by the staff throughout the intervention, and within-group support was provided by the patients themselves	T 25 (8%)/C 25 (8%) (b)	1.5 h × 6 w (9 h +homework)	POMS (tension/anxiety): *P* = 0.03 (between groups), *P* = 0.15 (group × time baseline, 6 weeks, 6 mos followup) HADS (anxiety) *P* = 0.40 between groups, *P* = 0.98 (group × time baseline, 6 weeks, 6 mos followup) (d)	Assessment of the effect on psychological distress indicated a significant decrease in total mood disturbance on the POMS over the study period	+

Deffenbacher et al., 1979 [61]	69 male/female students who scored in the upper 15% ile on the debilitating scale of the achievement anxiety scale Mean age: ND	Relaxation and breathing interventions/WLC/no treatment expectancy control	Relaxation as self-control involving discrimination training, relaxation training, application training, and guided practice in relaxation procedures. Modified desensitization involved learning relaxation as a coping skill, relaxation as self-control, and homework from relaxation skills learned, a scene presentation meant to relax the patient	Relaxation 17 (0%)/mod. desensitization 17 (12%)/control 17 (17%)/WLC 18 (0%) (b)	50 m × 7 (5.83 h) + homework	AAT D (debilitating anxiety) and AAT F (facilitating anxiety): posttest and followup between groups and two control groups *P* < 0.001; TAI: between groups at posttest *P* < 0.05 and at followup *P* < 0.001 (d)	Groups given relaxation as self-control and modified desensitization both reported significantly less debilitating test anxiety and significantly more facilitating test anxiety than controls. Relaxation as self-control group showed reduction and maintenance on both measures of nontargeted anxiety relative to the controls	+

Tables 3(a) and 3(b) have been split according to those programs that consist of “named” programs and those that consist of “un-named programs.” Because the un-named programs consist of sometimes multiple activities and are heterogeneous across, the authors have included the program description to complement those categories of studies.

AAT D: achievement anxiety test (debilitating anxiety); AAT F: achievement anxiety test (facilitating anxiety); BAI: Beck anxiety inventory; B-POMS: bipolar profile of mood states; BSI: brief symptom inventory; CAPS: clinician administered PTSD scale; BQ: Shirom-Melamed burnout questionnaire; CSQ: coping strategy questionnaire; DIS: dealing with illness scale; DSI: differential stress inventory; ELSS: everyday life stress scale; GHQ: general health questionnaire; HADS: hospital anxiety and Depression Scale; ISSL: inventory of stress symptoms lipp for adults; MCOS: measurement of current status; MESA: Measure for Assessment of General Stress Susceptibility; POMS: Profile of Mood States; PSS: Perceived Stress Scale; PTCI: Post-Traumatic Cognition Inventory; SCL-90: Symptom Checklist 90; SCL90-R (GSI); symptom checklist 90 global severity index; STAI: state-trait anxiety inventory; TAI: trait anxiety inventory; WSQP: work-related questionnaire for chronic psychiatric patients.

(a) Power calculation done and achieved, (b): power calculation not done or reported, (c) unclear if power calculation done or achieved, and (d) effect size not reported, ND: not described, WLC: wait list control, T: treatment, C: control.
